# Effects of Selenium-Enriched Rape Returning Amount on Available Selenium Content in Paddy Soil and Selenium Accumulation in Rice

**DOI:** 10.1155/2022/3101069

**Published:** 2022-12-17

**Authors:** Wei Huang, Qingguo Xu, Ning Wu

**Affiliations:** ^1^College of Agriculture, Hunan Agricultural University, Changsha, China; ^2^National Grain Industry (Functional Rice) Technology Innovation Center, Institute of Functional Agriculture, Yulin Normal University, Yulin, China; ^3^Key Laboratory of Beibu Gulf Offshore Engineering Equipment and Technology, Beibu Gulf University, Qinzhou 535000, China

## Abstract

Selenium-rich rape “*Selenium Ziyuan No*.1” was used as green manure to study the effects of different amounts of green manure returned to the field on the release characteristics of available selenium in acidic paddy soil in southern China, and to analyze the absorption and transformation of selenium in rice, so as to provide a theoretical basis for planting natural selenium-rich rice in acidic areas of southern China. Six treatments with different amounts of selenium-enriched rapeseed returning (0, 5, 10, 15, 20, and 25 t/hm^2^) were set up. Two rice varieties (selenium-rich rice variety Meixiangzhan 2 and common rice variety Zhongguangxiang 1) were selected. The results showed that (1) with the increase of selenium-rich rapeseed returning amount, the available selenium in soil showed an increasing trend. Over time, soil available selenium showed a significant increasing trend, and the content of soil available selenium reached the maximum at tillering stage, and then decreased. (2) For selenium-rich varieties, when the amount of selenium-rich rapeseed returned to the field was less than 15 t/hm^2^, the selenium content in rice grains increased significantly with the increase of the amount of selenium-rich rapeseed returned to the field, then remained basically stable. For conventional varieties, with the increase of the amount of selenium-rich rapeseed returned to the field, the selenium content of rice grains showed an increasing trend, but the overall selenium content was much lower than that of selenium-rich variety. (3) With the increase of the amount of rapeseed returned to the field, the rice yield had an increasing trend, but the maximum rice yields appeared when the amount of selenium-rich rapeseed returned to the field was 15 t/hm^2^. Therefore, Se-enriched rape returning could promote the release of available selenium in soil and the enrichment of selenium in rice plants, and significantly increase the selenium content in rice. According to the selenium content and yield of rice, it is suggested that the selenium-rich rice variety Meixiangzhan 2 was chosen and the amount of Se-rich rape returning is 15 t/hm^2^.

## 1. Introduction

Selenium is a trace element that the human body needs but cannot synthesize, and must be supplemented from the outside, and all organs of the human body need a certain amount of selenium to maintain its function [1]. Selenium deficiency to a certain extent will lead to hypertension, diabetes, coronary heart disease, asthma, and more than 40 kinds of acute and chronic diseases [2]. Selenium deficiency is an important reason for the high incidence of various cancers and chronic diseases [3]. Selenium in rice, vegetables, and fruits is the main source of selenium absorbed by the human body [4]. Selenium in rice is relatively stable and easy to preserve, which is regarded as an important source of selenium-rich food [5]. However, rice likes to absorb and accumulate heavy metal cadmium, which is not an essential element for the human body, and is harmful to human health [6]. Previous studies have shown that there is a significant negative correlation between selenium and cadmium in rice [7]. Increase in the selenium content in rice means reducing the content of cadmium, a heavy metal element. Because the selenium content of rice, vegetables, and fruits is relatively low under natural growth conditions, the traditional method is crop-added exogenous selenium by increasing selenium fertilizer or spraying selenium on the leaves [8–10]. This practice not only increases the economic cost but also has the risk of environmental pollution, which is not conducive to the safe production of agriculture [11].

Green manure is usually used to improve soil organic matter and selenium content, so as to increase the selenium content in rice. It can also be used as a high efficient material to active selenium in the soil [12]. In recent years, in order to improve the system of farmland rotation and fallow, the Ministry of Agriculture and Rural Affairs of China has jointly promoted this work with the Ministry of Finance every year. The implementation scale will be expanded to 240 million acres in 2021 [13]. In the south, the planting modes of *Rice-Rape* or *Rice-Rice-Rape* are mainly promoted [13]. As the selenium-rich rape is returned to the field, the soil organic matter is improved, and the activation of the soil selenium element is promoted. In addition, the selenium absorbed by the selenium-rich rape could be directly used as a source of activated organic selenium for rice to absorb and utilize. However, little was known about the effects of selenium-enriched rape returning on the release dynamics of soil available selenium, and the characteristics of absorption and accumulation of selenium in rice [14]. Therefore, it is of great significance to study the effects of the amount of selenium-rich rape returned to the field on the available selenium content in soil and the accumulation of selenium in rice.

At present, research on selenium-enriched rice is mainly reflected in the impact of selenium addition on rice yield and selenium content, while the research on activating selenium in soil to achieve natural selenium-enriched rice is relatively rare [12, 15]. Rape, as a kind of green manure, is one of the important traditional methods to improve the content of soil organic matter. It is a key measure to improve the soil and increase grain production. Turning over rape can increase the content of humus and large aggregates in soil, increase the ratio of loose and stable soil, increase the enrichment coefficient of >5 mm particle size carbon, and increase the contribution rate of aggregates to soil organic matter [16]. Rape rotation can increase the content of organic matter, total nitrogen, and alkali-hydrolyzable nitrogen in the soil surface, and intercropping population structure also plays a certain role in improving soil fertility [17]. According to Zhang's research [18], in terms of improving soil nutrient content, the effect of ploughing green manure rape is more significant, which not only reduces the amount of chemical fertilizer but also improves soil nutrient content and increase production.

There was a significant positive correlation between the amount of rapeseed returned to the field and the yield of rice and the number of soil bacteria, and a significant positive correlation between the amount of rapeseed returned to the field and the soil alkali-hydrolyzable N, available P, organic matter, pH, total number of culturable microorganisms, and *B*/*T* value [19]. The comprehensive metabolic activity of soil microbial carbon source was the strongest, the biodiversity was the highest, and Shannon index and evenness index are the highest in the treatment of the amount of rape returning is 15.0 t/hm^2^, which is more conducive to the stability of soil microecosystem [19]. According to Fan et al. [20], the return of rape to the field can increase the organic matter content and rice yield, mainly by increasing the soil dissolved organic carbon (DOC) and enhancing the activities of soil catalase and cellulase.

Wang et al. [21] investigated the correlation between soil and selenium-rich rice grains in the main rice-producing areas in eastern and southern Guangxi, and found that there was a significant positive correlation between soil organic matter content and selenium in paddy fields. Soil organic matter content plays a special role in the transportation of selenium between soil and plants, affecting the absorption and accumulation of selenium by plants, especially the selenium content in rice grains. The research of Han et al. [22] shows that such a rule also exists in dry land, and the change of soil organic matter can explain the variation of total selenium in surface soil >60%. This shows that under the same climatic conditions, the organic matter content of soil developed from similar parent material is the main factor affecting the total selenium content of soil [23–25]. However, there are also contrary views. Huang et al. [26] showed that soil available selenium was negatively correlated with soil organic matter and soil cation exchange capacity, and positively correlated with soil acidity and alkalinity. Selenium content in rice was positively correlated with available selenium and total selenium in soil. Different rice genotypes have significant differences in the enrichment of selenium in soil: Zhao et al. used the method of applying selenium fertilizer to soil in a pot experiment, and the selenium-rich genotype Wuyou 308 was 4.69 times the selenium content of the lowest variety [27]; Zhang et al. [28] collected 80 rice varieties and planted them in natural selenium-rich soil in Fengcheng City, Jiangxi Province, and evaluated the selenium content in brown rice. A series of selenium-rich rice varieties were screened out and used as high selenium-rich rice germplasm resources for the breeding of new selenium-rich rice varieties. According to Zhang et al.'s research [29], rice genotype and the proportion of available selenium in soil are the main factors for the accumulation of organic selenium in rice grains. However, the distribution of selenium in different parts of different rice genotypes was completely consistent, which was root > stem and leaf > grain. Selenium content in different parts of rice and organic selenium content in brown rice increased with the increase of the soil selenium application level. The selenium content of high accumulation rice varieties was significantly higher than that of low accumulation rice varieties.

So far, there are many studies on using green manure to return to the field to improve soil organic matter, thereby increasing the absorption of selenium by rice. However, there is no relevant paper on the use of selenium-rich rapeseed returned to the field to improve soil organic matter content, and also to directly improve soil available selenium content without artificial application of selenium fertilizer. The release process of available selenium in soil, the distribution law of selenium in rice plants, and its effect on yield need to be further studied after the selenium-rich rape was returned to the field.

In the blooming stage of selenium-rich rape, after picking the flowering Chinese cabbage, the aboveground part of rape was cut and directly returned to the field after cutting. The release dynamics of available selenium in soil and the enrichment characteristics of selenium in roots, stems and leaves, and grains of different rice varieties under different amounts of selenium-rich rape returning were analyzed, and the appropriate amount of rape returned to the field was determined according to the selenium content of rice in each treatment. At the same time, the corresponding field demonstration and technology promotion work were carried out to provide theoretical basis and technical support for the large-scale production of selenium-rich rice.

## 2. Materials and Methods

### 2.1. Experimental Site and Soil Characterization

**Figure 1:**
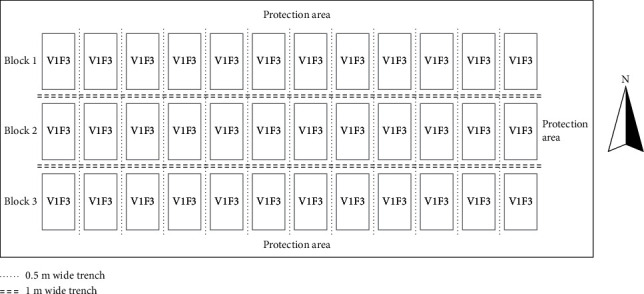
Layout plan of the rice planting community.

The experiment was carried out in the rice planting base of Zhangmu Town, Fumian District, Yulin City, Guangxi, from December 2020 to July 2021. Before the experiment, the tested paddy field was a typical selenium-rich soil, with available selenium content of 83.89 *μ*g/kg and total selenium content of 1.07 mg/kg. Other basic physical and chemical properties were as follows: pH is 5.18, total nitrogen content is 1.98 g/kg, total phosphorus content is 1.15 g/kg, total potassium content is 1.23 g/kg, alkali-hydrolyzable nitrogen content is 89.27 mg/kg, available phosphorus content is 44.06 mg/kg, and the content of organic matter is 31.29 mg/kg.  Figure 1 shows the layout plan of the rice planting community.

### 2.2. Test Materials

Selenium-rich rapeseed variety *Selenium Ziyuan No*.1 was selected as green manure in winter fallow field. *Selenium Ziyuan No*. 1 was cultivated by Wang Hanzhong, a member of the Chinese Academy of Engineering, and was the first new hybrid rapeseed variety in the world. The seeds were sown on December 10, 2020, and the seeding rate was 30 kg/hm^2^. After that rape was harvest on March 10, 2021, the overground part of the rape was cut, the green manure stems and leaves are cut into the length of 10∼20 cm, and then, the green manure stems and leaves are scattered on the ground and ploughed into surface soil about 20 cm deep. Irrigation and retting was done first, and then transplanting of rice seedlings on April 10.

### 2.3. Methods

#### 2.3.1. Test Design

Six amounts of rapeseed returned to the field, and two rice varieties were set up. Six amounts of selenium-rich rape returned to the field were F1 (0 t/hm^2^), F2 (5 t/hm^2^), F3 (10 t/hm^2^), F4 (15 t/hm^2^), F5 (20 t/hm^2^), and F6 (25 t/hm^2^). Two rice varieties were selenium-rich rice variety V1 (Meixiangzhan 2) and common rice variety V2 (Zhongguangxiang 1), where F1 is set to CK. Meixiangzhan 2 was bred by the Rice Research Institute of Guangdong Academy of Agricultural Sciences. Zhongguangxiang 1 was bred by the Institute of Crop Science, Chinese Academy of Agricultural Sciences. There were 12 treatments, and each treatment was repeated 3 times. Randomized block arrangement was used in the experiment, and each plot was 12 m long, 5 m wide, and 60 m^2^ in area. The experimental plots were separated by ridges and ditches with a width of 50 cm left to avoid cross influence. The water and fertilizer management, pest control, and other field management work after transplanting are the same as the traditional methods.

After being cut, the selenium-rich rape was weighed according to the design requirements and the area of the test area, and then moved to each test area to be turned over and returned to the field. Soil samples were collected from each test area by the five-point sampling method on March 10 (D1: the day rape returned to the field), April 10 (D2: the day rice was transplanted), May 8 (D3: tillering stage), May 26 (D4: jointing stage), June 12 (D5: poplar flower heading stage), and July 28 (D6: maturity stage) in 2021. Then, they measure the available selenium content of the soil. On July 28, rice yield was measured, and rice plants were collected. Then, the content of selenium in rice roots, stems and leaves, and grains was measured.

#### 2.3.2. Determination Items and Methods

The available selenium content of the tested soil was measured by the NaH_2_PO_4_ extraction method [30]. After rice roots, stems and leaves, and grains are dried and crushed, the total selenium content is determined according to the National Food Safety Standard Determination of selenium in Food (GB 5009.93-2017) [31]. The method of rice yield measurement is based on the National Measures for Acceptance of Grain Yield Measurement for High Yield Establishment (Trial) issued by the General Office of the Ministry of Agriculture of the People's Republic of China in June, 2008 [32].

#### 2.3.3. Statistical Analysis

The experimental data were analyzed by two factor analysis of variance and was used in IBM SPSS Statistics 22 software, and the model was designated as the interaction between the variety and the amount of rape returned to the field [33]. The main effects were compared, and the confidence interval was adjusted by LSD method. The significance level was 0.05, and the confidence interval was 95%. Duncan's method was used to compare the differences in one-way analysis of variance. WPS Office 2021 was used to sort out the test data and make charts.

## 3. Results

### 3.1. Available Selenium in Soil

It can be seen from Table 1 that the effect of each treatment on the soil available selenium content on the day of rape returning to the field was not significant (0.05 level). There was no significant difference in available selenium content of soil sampled at different time in F1 treatment. The return amount of selenium-enriched rape (F2–F6) had a significant effect on the soil available selenium content at other sampling times (0.01 level).

**Figure 2:**
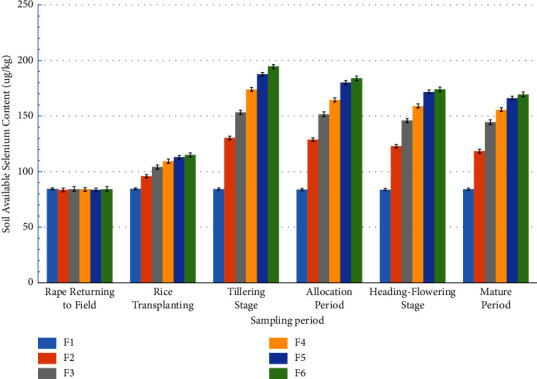
Effect of rape returning amount on available selenium content in soil.

It can be seen from Figure 2 that with the increase of the amount of selenium-rich rape returned to the field, the available selenium in the soil showed an increasing trend. On the day of rice transplanting, the available selenium content of F2, F3, F4, F5, and F6 significantly increased by 13.73%, 23.36%, 29.51%, 33.79%, and 36.12%, compared with F1, respectively. The difference between F5 and F4, F5 and F6 was not significant, and the difference between other treatments was significant. At the tillering stage, the available Se content of F2, F3, F4, F5, and F6 increased significantly by 54.60%, 82.12%, 106.48%, 122.97%, and 130.98%, compared with F1, respectively. At the jointing stage, the available Se contents of F2, F3, F4, F5, and F6 were 53.67%, 80.85%, 96.30%, 114.81%, and 119.47% higher than that of F1, significantly. The differences between treatments were significant except between F5 and F6. The available Se content of F2, F3, F4, F5, and F6 increased, respectively, by 44.88%, 72.21%, 87.59%, 102.34%, and 105.08% compared with F1 at the heading stage of poplar. The differences between treatments were significant except between F5 and F6. At maturity, compared with F1, the available Se content of F2, F3, F4, F5, and F6 increased by 40.62%, 71.88%, 84.99%, 97.35%, and 101.41%, respectively, and the difference was significant. The differences between each treatment were significant except between F5 and F6.

It can also be seen from Figure 2 that when the amount of selenium-rich rape returned to the field is 0, there is no significant difference in the soil available selenium content in different periods. For other treatments, the soil available selenium showed a significant increasing trend with the passage of time, and the soil available selenium content reached the maximum at the tillering stage, and then showed a decreasing trend [13]. When the amount of selenium-rich rape returned to the field was 5 t/hm^2^, the contents of available Se in soil increased significantly by 14.80%, 55.87%, 54.20%, 47.05%, and 41.67% on D2, D3, D4, D5, and D6, respectively, compared with D1. There was no significant difference in soil available selenium content between D3 and D4, D5 and D6, and there was significant difference in the soil available selenium content in other periods [34]. When the amount of selenium-enriched rape returned to the field was 10 t/hm^2^, compared with D1, the available selenium content of D2, D3, D4, D5, and D6 increased by 23.57%, 82.23%, 80.09%, 73.45%, and 71.84%, significantly. There was no significant difference in soil available selenium content between D3 and D4, D4 and D5, D5 and D6, but there was significant difference in the soil available selenium content in other treatments [35]. When the amount of selenium-enriched rape returned to the field was 15 t/hm^2^, the available selenium content in soil increased by 30.12%, 107.24%, 96.09%, 89.54%, and 85.52% in D2, D3, D4, D5, and D6, respectively, compared with D1, and the differences were significant. There was no significant difference in the soil available selenium content between D3 and D4. There was also no significant difference in the soil available selenium content between D4 and D5, D6, but there was significant difference in soil available selenium content in other periods [20]. When the amount of selenium-enriched rape returned to the field was 20 t/hm^2^, compared with D1, the available selenium content in soil increased significantly by 35.03%, 124.79%, 115.53%, 105.35%, and 98.79% in D2, D3, D4, D5, and D6, respectively [6]. When the amount of selenium-rich rape returned to the field was 25 t/hm^2^, the available Se content in soil increased by 36.38%, 131.17%, 118.61%, 106.62%, and 101.41% on D2, D3, D4, D5, and D6, respectively, compared with D1. There was no significant difference in soil available selenium content between D5 and D6, but there was significant difference in soil available selenium content in other periods.

### 3.2. Selenium Distribution in Rice

It can be seen from Table 1 that the return amount of selenium-rich rape and rice varieties had significant effects on the selenium content in the rice roots, stems and leaves, and grains, but the interaction between the rice varieties and the return amount of selenium-rich rape only had a very significant effect on the selenium content in grains (0.01 level), a certain effect on the content of selenium in stems and leaves (Sig. 0.053).

#### 3.2.1. Root

**Figure 3:**
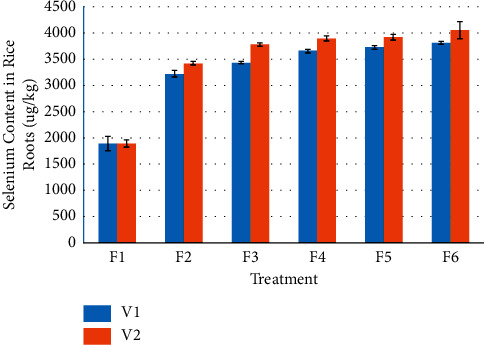
Effects of Se-enriched rape returning amounts on selenium content in rice root.

It can be found out from Figure 3 that compared with the selenium-rich variety V1, the root selenium content of V2 has an increasing trend. Compared with V1, the root selenium content of V2 significant increased by 9.92% and 6.32%, respectively, when the amount of selenium-enriched rape returned to the field was F3 and F6.

It can also be seen from Figure 3 that with the increase of the amount of rape returned to the field, the selenium content in rice roots has an increasing trend. For selenium-rich variety V1, the root selenium content of F2, F3, F4, F5, and F6 increased significantly by 70.35%, 81.73%, 93.52%, 97.05%, and 101.67%, respectively, compared with F1. There was no significant difference between F2 and F3, and among F4, F5, and F6, while there was significant difference among other treatments. For V2, compared with F1, the Se contents in roots of F2, F3, F4, F5, and F6 were increased by 80.61%, 99.39%, 105.55%, 106.96%, and 114.03%, respectively. The differences among F3, F4, and F5 were not significant, and the differences among F4, F5, and F6 were also not significant, while the differences among other treatments were significant.

#### 3.2.2. Stem and Leaf

**Figure 4:**
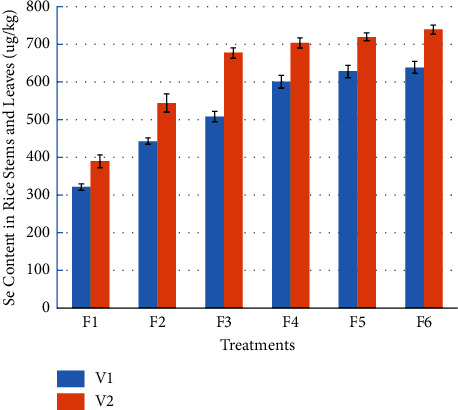
Effects of rape returning amounts on Se content in rice stems and leaves.

It can be seen from Figure 4 that compared with selenium-rich variety V1, the selenium content in stems and leaves of general variety V2 has an increasing trend. Compared with V1, the selenium content in the stems and leaves of V2 increased by 21.26%, 22.64%, 33.20%, 17.32%, 14.66%, and 15.69%, respectively, when the selenium-enriched rape returned to the field for F1 to F6, and the differences were significant.

It can also be seen from Figure 4 that with the increase of the amount of rape returned to the field, the selenium content in rice stems and leaves has an increasing trend. For selenium-rich variety V1, compared with F1, the stems and leaves selenium content of F2, F3, F4, F5, and F6 increased significantly by 37.88%, 58.15%, 86.75%, 95.34%, and 98.70%, respectively. The differences among treatments F4, F5, and F6 were not significant, while the differences among other treatments were significant. For the common variety V2, the Se contents in stems and leaves of F2, F3, F4, F5, and F6 increased by 39.46%, 73.72%, 80.69%, 84.71%, and 89.58%, respectively, compared with F1. The differences among F3, F4, and F5 were not significant, and the differences among F4, F5, and F6 were also not significant, while the differences among other treatments were significant.

#### 3.2.3. Grain

**Figure 5:**
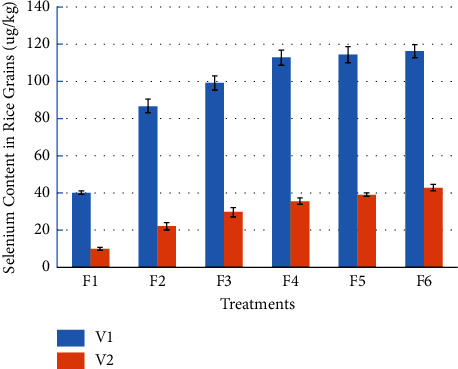
Effects of rape returning amounts on Se content in rice grain.

It could be made out from Figure 5 that compared with the selenium-rich variety V1, the grain selenium content of V2 has an obviously decreasing trend. The grain selenium content of V2 significantly decreased by 21.26%, 22.64%, 33.20%, 17.32%, 14.66%, and 15.69%, compared with V1, respectively, when the selenium-rich rapeseed returned to the field for F1 to F6.

It can also be seen from Figure 5 that with the increase of the amount of rape returned to the field, the selenium content of rice grains has an increasing trend. For selenium-rich variety V1, the root selenium content of F2, F3, F4, F5, and F6 significantly increased by 115.52%, 146.66%, 180.58%, 184.05%, and 189.18%, compared with F1, respectively. For V2, the selenium content in F2, F3, F4, F5, and F6 increased significantly by 123.17%, 198.63%, 258.13%, 293.13%, and 330.57%, compared with F1, respectively. There was no significant difference between F2 and F3, between F3 and F4, and among F4, F5, and F6.

From Table 1, we can also see that for the grain selenium content, there is an interaction between the rice variety and the amount of selenium-rich rape returned to the field. Figure 5 also shows that for the selenium-rich varieties, when the amount of selenium-rich rapeseed returned to the field is less than 15 t/hm^2^ (F4), the selenium content of rice grains increases significantly with the increase of the amount of selenium-rich rapeseed returned to the field. The selenium content in rice grain remained stable during the exceeds of this rape returning amount. For V2, with the increase of the amount of selenium-rich rapeseed returned to the field, the selenium content of rice grains showed an increasing trend, but the overall selenium content was much lower than that of the selenium-rich variety V1.

### 3.3. Rice Yield

**Figure 6:**
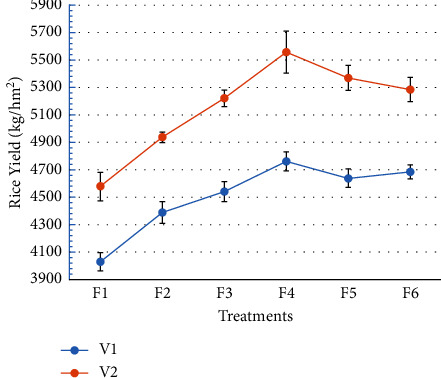
Response of rice yield to amounts of selenium-enriched rape returning.

It can be seen from the rice yield data in Table 1 that rice varieties and the amount of selenium-rich rapeseed returned to the field have significant effects on rice yield, but the interaction between them has no significant effect on yield. It can be perceived from Figure 6 that the rice yield of V2 increased significantly by 13.63%, 12.53%, 14.98%, 16.74%, 15.79%, and 12.85% compared with V1 from F1 to F6.

It can also be seen from Figure 6 that with the increase of the amount of rape returned to the field, the yield of rice has an increasing trend. For selenium-rich variety V1, the root selenium content of F2, F3, F4, F5, and F6 increased significantly by 8.93%, 12.72%, 18.16%, 15.12%, and 16.26% compared with F1, respectively. The difference between F2 and F3 was not significant, the difference among F3, F4, F5, and F6 was also not significant, and the difference among other treatments was significant. The maximum yield was reached to 4758.38 kg/hm^2^ in V1F4. For V2, compared with F1, the yields of F2, F3, F4, F5, and F6 were significantly increased by 7.87%, 14.05%, 21.39%, 17.31%, and 15.47%, respectively. There was significant difference except between F4 and F5, and among F3, F5, and F6. The maximum yield was 5554.92 kg/hm^2^ in V2F4.

## 4. Discussion

It is generally recognized that the application of green manure can improve soil organic matter and soil properties. In this study, there are three reasons for adopting “*Selenium Ziyuan No*.1” rape. First, rape is a kind of green manure; second, the soil phosphorus content in Fumian area of Yulin City is very high, and planting rape can absorb a lot of phosphorus; finally, “*Selenium Ziyuan No*.1” rape is a selenium-rich variety, which can effectively absorb selenium in soil and achieve natural selenium enrichment without adding extra selenium. The factors of applying green manure include the available selenium in soil, the selenium content in rice, and the resulted rice yield, which will be discussed in detail in the following section.

### 4.1. Available Selenium in Soil

Previous studies have shown that the return of rape as green manure can increase soil dissolved organic carbon (DOC), enhance soil catalase and cellulase [20], increase the content of humus and large aggregates in soil, increase the ratio of soil tightness and stability [16], increase the comprehensive metabolic activity of soil microbial carbon source, and improve biodiversity index [19] so as to increase the organic matter content of the soil surface [17, 20], which could increase the yield of rice [19, 20]. Organic matter is the main factor affecting the activity of soil available selenium, and there is a significant positive correlation between soil selenium activity and soil organic matter content [21, 22]. This is consistent with the results of this paper. The research in this paper shows that the amount of selenium-rich rape returned to the field had a significant effect on the available selenium content of the soil. With the increase of the amount of selenium-rich rape returned to the field, the available selenium in the soil showed an increasing trend. At the same time, the return of selenium-rich rape to the field not only activates soil available selenium by increasing soil organic matter but also directly provides natural selenium sources.

This study also showed that the amount of selenium-rich rapeseed returned to the field affected the available selenium content in the soil. With the passage of time, the available selenium content in the soil showed a significant increasing trend, reaching the maximum at the tillering stage, and then decreased slowly. The release of the selenium element of the selenium-rich rape after being pressed green and returned to the field is affected by the decomposition degree. Until the day of rice transplanting, the increase of soil available selenium content was relatively limited in all treatments, indicating that the decomposition of rape plants was slow, which may also be related to the low temperature during spring ploughing. After rice transplanting, with the temperature rose, the decomposition rate of rape was accelerated, and a large number of organic matter and selenium elements were released into the soil, so the available selenium content of the soil increased rapidly and reached its peak at the tillering stage. After that, the remaining rape residues were relatively few. Coupled with the absorption of rice roots, the soil available selenium content began to decline. Therefore, it can be inferred that rape is easy to decay in the soil, but the release of selenium is mainly concentrated in 30∼60 days after ploughing, which is basically consistent with the previous research results on the decay law of rape after returning to the field and the release characteristics of N, P, and K [2, 36, 37].

However, some people hold the opposite view. Han et al. [22] deemed that soil available selenium was negatively correlated with soil organic matter and soil cation exchange capacity. The possible reason for the result is that the soil properties of the two places are really different: Shahe County is located in the central of China where the soil is mainly transformed from alluvial deposits of modern rivers and lake sediments in the southeast plain lake area, with deep soil layer, moderate texture, good ventilation, permeability, fertilizer supply, strong water and fertilizer retention, high organic matter content, and slight acidity. Located in the south of China, Fumian region is a typical acid red soil distribution area with thin soil layer and high viscosity. Meanwhile, with the aeration, water permeability, and fertilizer supply are very poor, the soil organic matter content is very low, and the soil is acidic.

### 4.2. Selenium Content in Rice

Previous studies have shown that rice genotype and the proportion of available selenium in soil are the main factors for the accumulation of selenium in rice grains [25]. However, the distribution of selenium in different parts of different rice genotypes was completely consistent, which was root > stem and leaf > brown rice. The selenium content of each part of rice and the content of organic selenium in brown rice increased with the increase of selenium application level, and the selenium content of high enrichment rice varieties was significantly higher than that of the low enrichment rice varieties [29, 34]. This is consistent with the results of this paper. The study showed that the effects of rice varieties and the amount of selenium-rich rape returned to the field on the selenium content in roots, stems and leaves, and grains of rice were significant. Compared with the selenium-rich variety Meixiangzhan 2, Zhongguangxiang 1 had a tendency to increase the selenium content in roots and stems and leaves, but the selenium content in grains decreased significantly.

Tang used milk vetch as green manure, and the study showed [29] that when the amount of green manure returned to the field was insufficient, the selenium supply in the soil was limited, and the selenium content in rice hovered at 0.032–0.036 mg/kg, which was difficult to achieve a breakthrough increase. With the increase of the amount of green manure returned to the field, the grain selenium content and yield of rice showed an increasing trend. At 22.5–24 t/hm^2^, the selenium content of rice increased to 0.044–0.047 mg/kg, which meets the standard of selenium-rich rice in China (GB/T2499-2008). This is not consistent with the research in this paper. The results showed that the selenium content in rice grain increased significantly with the increase of green manure application. When the amount of green manure application reached 15 t/hm^2^, the selenium content in rice grains remained basically stable even if it exceeded this amount. When the amount of green manure used is more than 5 t/hm^2^, the selenium content of rice grains reached the standard of selenium-rich rice. The possible reasons for the result are as follows: first, the differences between different rice varieties. Second, the difference between the effects of different kinds of green manures [38]. Therefore, selenium-rich rape, as a kind of green manure, plays an important role in the process of soil selenium activation.

The study also showed that there was an interaction between the rice varieties and the amount of selenium-enriched rapeseed returned to the field. For selenium-enriched rice variety Meixiangzhan 2, the selenium content in rice grains increased significantly with the increase of the amount of selenium-enriched rapeseed returned to the field when the amount was less than 15 t/hm^2^, and remained stable when the amount was more than 15 t/hm^2^. For Zhongguangxiang 1, with the increase of the amount of selenium-rich rape returned to the field, the selenium content in rice grains increased, but the overall selenium content was much lower than that of the selenium-rich variety Meixiangzhan 2.

### 4.3. Rice Yield

Returning green manure rape to the field can increase soil pH value, alleviate soil acidification, increase total porosity of paddy soil, reduce bulk density, improve soil's physical properties [39], improve soil fertility, increase the number of grains per panicle and effective panicles, increase rice yield, improve rice appearance quality and processing quality, and improve nutritional quality and steaming quality [35, 40]. At the same time, rape returning to the field also has a certain inhibitory effect on planthoppers and sheath blight [41]. According to Zhou's et al. research [19], when the rape “Huyou 17” returning is 15–22.5 t/hm^2^, the rice yield is significantly increased. Especially when the amount of returning to the field was 15 t/hm^2^, the comprehensive metabolic activity of soil microbial carbon source in the treatment of returning rape to the field was the strongest, and the yield of rice “Qingxiang Ruanjing” reached the maximum. This is consistent with the results of this paper. The results showed that the rice yield increased with the increase of the amount of rapeseed returned to the field, but the maximum yield appeared when the amount of selenium-rich rapeseed returned to the field was 15 t/hm^2^.

Zhu et al. [42] held that after the rape “Huyou 21” was returned to the field as green manure, the contents of alkali-hydrolyzable nitrogen, available phosphorus, and available potassium in the soil were higher than those in the field without green manure, the plant height and chlorophyll content of rice “Qingxiangruanjing” increased, and the yield of rice increased, with the increase of rape returned to the field. This is basically consistent with the results in this paper. However, he believed [42] that when the amount of rapeseed returning reached 22.5 t/hm^2^, the rice yield reached the maximum. The main reasons for the difference were as follows: first, the difference between different rape varieties; second, the differences of soil properties in different regions; finally, and perhaps most importantly, the test site is located in Guangxi. The temperature is higher than the experimental field of Zhu's, and the high temperature promotes the decomposition of rape returned to the field and improves the efficiency of rape returned to the field [35, 38].

There are differences in the yield of different rice varieties, which is the driving force of human breeding efforts [43]. The study showed that the yield of selenium-rich rice varieties was lower than that of common rice varieties. This indicates that the enrichment of selenium in rice gain is at the expense of yield, and it is difficult to achieve the maximum level at the same time for both yield and selenium content. This study also showed that although there were differences in yield among the different rice varieties, the trend of yield difference was the same for the same amount of selenium-rich rapeseed returning.

## 5. Conclusion

The results show that (1) with the increase of selenium-rich rapeseed returning amount, the available selenium in soil showed an increasing trend. Over time, soil available selenium showed a significant increasing trend, and the content of soil available selenium reached the maximum at the tillering stage, then decreased. (2) For selenium-rich varieties, when the amount of selenium-rich rapeseed returned to the field was less than 15 t/hm^2^, the selenium content in rice grains increased significantly with the increase of the amount of selenium-rich rapeseed returned to the field, then remained basically stable. For conventional varieties, with the increase of the amount of selenium-rich rapeseed returned to the field, the selenium content of rice grains showed an increasing trend, but the overall selenium content was much lower than that of the selenium-rich variety. (3) With the increase of the amount of rapeseed returned to the field, the rice yield had an increasing trend, but the maximum rice yields appeared when the amount of selenium-rich rapeseed returned to the field was 15 t/hm^2^.

Therefore, Se-enriched rape returning could promote the release of available selenium in soil and the enrichment of selenium in rice plants, and significantly increase the selenium content in rice. According to the selenium content and yield of rice, it is suggested that the selenium-rich rice variety Meixiangzhan 2 was the choice and the amount of selenium-rich rape returned to the field is 15 t/hm^2^ [44].

## Figures and Tables

**Table 1:** Effect test between subjects.

Sources	Dependent variables	Type III sum of squares	df	Mean squares	*F*	Sig.
*V*	SoilSe1	0.67	1	0.667	0.078	0.783
	SoilSe2	0.31	1	0.314	0.021	0.885
	SoilSe3	1.85	1	1.854	0.066	0.799
	SoilSe4	3.42	1	3.423	0.055	0.816
	SoilSe5	50.93	1	50.932	1.195	0.285
	SoilSe6	14.87	1	14.874	0.743	0.397
	RootSe	367973.67	1	367973.670	23.179	<0.001
	StemSe	100245.06	1	100245.058	152.949	<0.001
	GrainSe	37994.46	1	37994.456	1628.866	<0.001
	Yield	3819412.26	1	3819412.263	188.971	<0.001

YC	SoilSe1	4.31	5	0.861	0.100	0.991
	SoilSe2	4025.41	5	805.083	55.030	<0.001
	SoilSe3	51601.33	5	10320.267	369.556	<0.001
	SoilSe4	42462.75	5	8492.551	137.671	<0.001
	SoilSe5	35619.15	5	7123.829	167.193	<0.001
	SoilSe6	32367.00	5	6473.400	323.398	<0.001
	RootSe	17536241.83	5	3507248.366	220.929	<0.001
	StemSe	502254.60	5	100450.920	153.263	<0.001
	GrainSe	12579.27	5	2515.855	107.858	<0.001
	Yield	2818603.92	5	563720.784	27.891	<0.001

*V∗*YC	SoilSe1	6.17	5	1.234	0.144	0.980
	SoilSe2	3.43	5	0.685	0.047	0.999
	SoilSe3	21.78	5	4.356	0.156	0.976
	SoilSe4	117.49	5	23.498	0.381	0.857
	SoilSe5	65.47	5	13.094	0.307	0.904
	SoilSe6	6.07	5	1.214	0.061	0.997
	RootSe	91858.95	5	18371.791	1.157	0.359
	StemSe	8422.19	5	1684.437	2.570	0.053
	GrainSe	2322.76	5	464.552	19.916	<0.001
	Yield	77544.22	5	15508.844	0.767	0.582

## Data Availability

The data that support the findings of this study are available from the corresponding author upon reasonable request.
